# Do fully erupted third molars affect the dental and periodontal health of adjacent second molars? A CBCT-based retrospective study in the Yemeni population

**DOI:** 10.1186/s12903-025-06667-y

**Published:** 2025-08-11

**Authors:** Manal M. Al-Hajri, Abduljabbar M. Al-Sameai

**Affiliations:** Department of Oral Medicine, Oral Diagnosis, Periodontology and Oral Radiology, Faculty of Dentistry, Sana’a University, Sana’a, Yemen

**Keywords:** Caries, Cone-beam computed tomography, Effect, Erupted third molar, Periodontal condition, Second molar, Yemen

## Abstract

**Background:**

Periodontal pathologies and caries affecting second molars (M2s) are frequently linked to the presence of adjacent third molars (M3s). This study assessed the association between fully erupted M3s and dental and periodontal/cariogenic conditions in M2s using cone-beam computed tomography (CBCT).

**Methods:**

A retrospective comparative analysis evaluated 400 CBCT scans from Yemeni patients (≥ 18 years) collected between January 2019 and March 2021. Scans were divided equally into Group I (with fully erupted M3s, *n* = 200) and Group II (without M3s, *n* = 200). Outcomes included caries, root canal treatment (RCT), external root resorption (ERR), furcation involvement (FI), lamina dura (LD) integrity, and alveolar bone resorption (ABR) at six anatomical sites around each second molar. Data were analyzed using SPSS version 24, with chi-square test for associations, Mann-Whitney test for mean comparisons, and a multivariable logistic regression model to determine adjusted odds ratios (AORs) and confidence intervals (CIs), considering a p-value < 0.05 as statistically significant.

**Results:**

Fully erupted M3s significantly increased risks of adjacent M2 pathologies (all *p* < 0.001). Group I exhibited markedly higher adjusted odds of caries (AOR = 32.53), ERR (AOR = 6.26), FI (AOR = 19.99), RCT (AOR = 9.15), and LD absence (AOR = 10.72) compared to Group II. ABR at all anatomical sites (mesiobuccal, mesiolingual, mid-buccal, mid-lingual, distobuccal, distolingual) was greater in Group I (*p* < 0.05).

**Conclusions:**

Fully erupted M3s are strongly associated with periodontal and cariogenic complications in adjacent M2s within the Yemeni population. CBCT proves effective for diagnosing structural periodontal pathologies, underscoring its utility in clinical evaluations.

## Introduction

Periodontal diseases and dental caries represent the most prevalent noncommunicable diseases in humans, with rising global incidence [1]. These conditions severely compromise quality of life, self-esteem, and nutritional status through tooth loss and chronic pain. Dental biofilm serves as a major biological determinant driving both pathologies [1–3]. Periodontitis is “a chronic multifactorial inflammatory disease associated with symbiotic plaque biofilms and characterized by progressive destruction of the tooth-supporting apparatus” [4]. Periodontitis and dental caries are prevalent mostly in the area of third molars (M3s) [5], increasing their exposure to periodontal infection and causing greater breakdown of periodontal tissues. Moreover, maintaining proper oral hygiene in posterior areas is relatively difficult, which possibly causes plaque accumulation that may affect the second molar (M2) [5, 6].

Some obstacles often cause M3s to be held back during eruption, including inadequate arch space, pathological conditions, or anatomical barriers including proximity to the mandibular ascending ramus, inadequate buccal alveolar bone thickness, or relationship to the infratemporal fossa [7, 8], making this area difficult to clean. Furthermore, enclosed or embedded M3s promote dental plaque accumulation, thus increasing the risk of developing infectious diseases in molar areas [9]. The presence of caries and periodontal disease distal to M2 is often associated with the topographic status of M3 [10]. Severe pathology involving both teeth may necessitate extraction of M2 and M3 [11], confirming M3s as significant threats to M2 survival.

Several studies have investigated the potential association between asymptomatic M3s and periodontal damage to the distal aspect of M2s [12, 13]. Even without periodontal symptoms, periodontal damage to the distal aspect of M2s can also be present [14]. In addition, M3s may continue to have a negative impact on M2 health well into later life [6]. Crucially, the specific influence of fully erupted asymptomatic M3s on the periodontal and cariogenic status of adjacent M2s remains largely unknown [15, 16], creating a significant knowledge gap.

Clarifying this relationship has direct implications for clinical decision-making regarding M3 management. Evidence-based determination of whether fully erupted M3s pose significant risks to adjacent teeth will inform guidelines on prophylactic extraction versus long-term retention. This is especially critical for preventive strategies targeting M2 preservation, as early intervention could mitigate complex treatments like root canals, resorption management, or regenerative periodontal surgery. Establishing concrete associations would transform radiographic findings into actionable clinical protocols for monitoring and intervention.

Therefore, this CBCT-based study aimed to definitively evaluate the association between fully erupted M3s and M2 dental caries, root canal treatment (RCT), and periodontal pathologies, including external root resorption (ERR), furcation involvement (FI), lamina dura (LD), alveolar bone resorption (ABR), while assessing demographic modifiers (sex, side, and age).

This investigation holds particular urgency within the Yemeni population, where compromised periodontal health and localized periodontitis are exacerbated by socioeconomic challenges, suboptimal oral hygiene practices [17], and high prevalence of risk factors like smoking and khat chewing [18]. As the first Yemeni study to radiographically assess the effect of fully erupted M3s on adjacent M2 health using CBCT, its findings provide crucial population-specific evidence to guide clinical practice and preventive oral healthcare strategies in this underserved region.

## Materials and methods

### Study design and setting

This was a retrospective radiographic comparative study. CBCT scans of the maxilla and/or mandible were obtained from private radiology centers in Sana’a, Ibb, and Aden, referred for diagnostic purposes such as implant planning, endodontic assessment, and third molar evaluation from January 2019 to March 2021.

### Study population and sampling

The study population comprised all CBCT scans archived within the targeted private radiology centers, totaling 8,350 images featuring either the presence or absence of third molars (M3s). According to OpenEpi [19], the minimum sample size required for this study was determined to be 384 individuals. To enhance statistical power and improve precision in representing population characteristics [20], the final study sample size was increased to 400. The study sample was randomly selected, adhering to specific inclusion and exclusion criteria to ensure balanced representation across the dental arches. Only CBCT images of patients aged 18 years and older with fully erupted M2s and either fully erupted or absent M3s were included. Conversely, we excluded images that were incomplete or of poor quality, and those featuring partially erupted molars, maxillofacial cysts or tumors in the molar area, jaw trauma or fracture, or less than two-thirds of root formation of M3s. The final sample consisted of two equal groups: Group I, which included cases with M3s (*n* = 200), and Group II, which included cases without M3s (*n* = 200).

### CBCT imaging protocol

All scans were acquired with a PaX‑i3D Green unit (Model PHT-60CFO, VATECH, Seoul, South Korea). Scans were performed at 95 kVp and 9 mA with an exposure time of 18.817 s, utilizing a 0.18 mm isotropic voxel resolution and field of view (FOV) of 10 × 11 cm to ensure comprehensive capture of the posterior dentition. DICOM datasets were reconstructed and analyzed using manufacturer-specific Ez3D-i software (Version 2.1.2, VATECH) in a controlled low-ambient lighting environment. All CBCT images were stored on an external hard disk. The data collection process took a period of 5 months, from June to October 2021.

### Variables

**Fig. 1:**
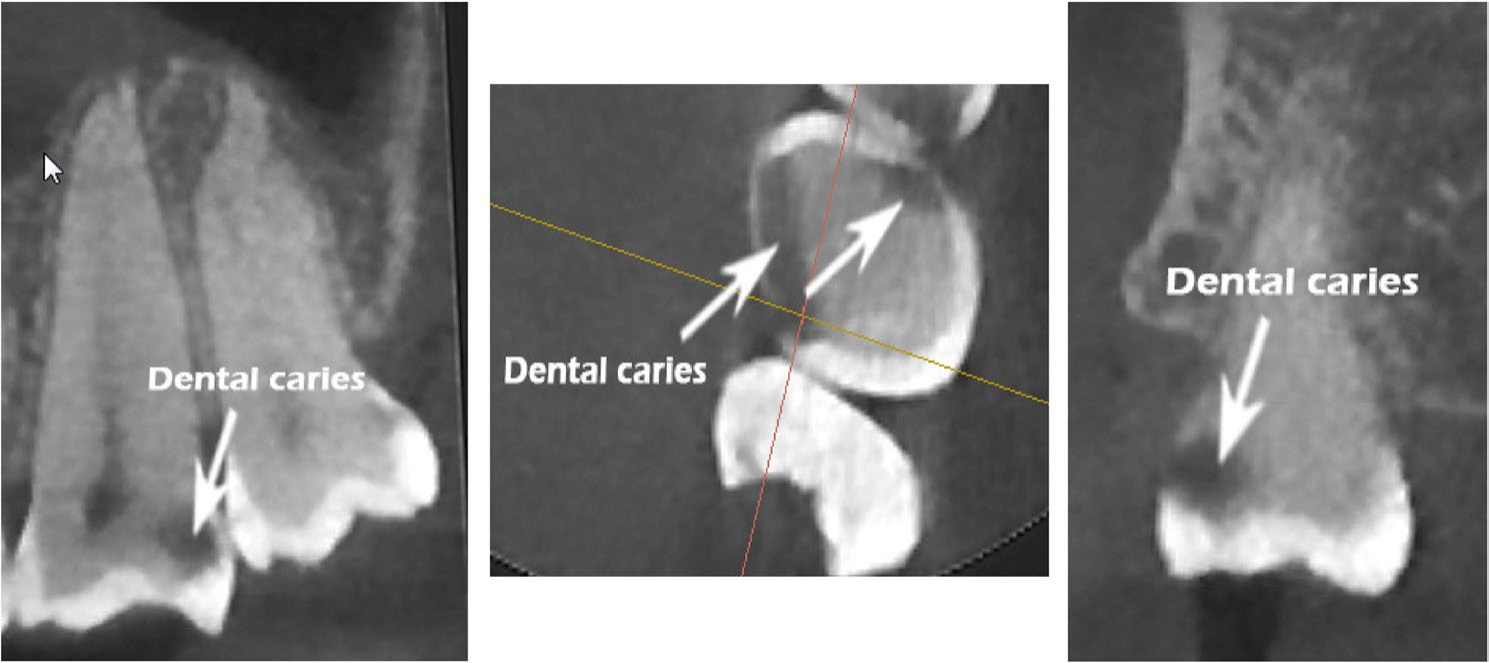
CBCT images showing dental caries of M2 in sagittal, axial, and coronal views

**Fig. 2:**
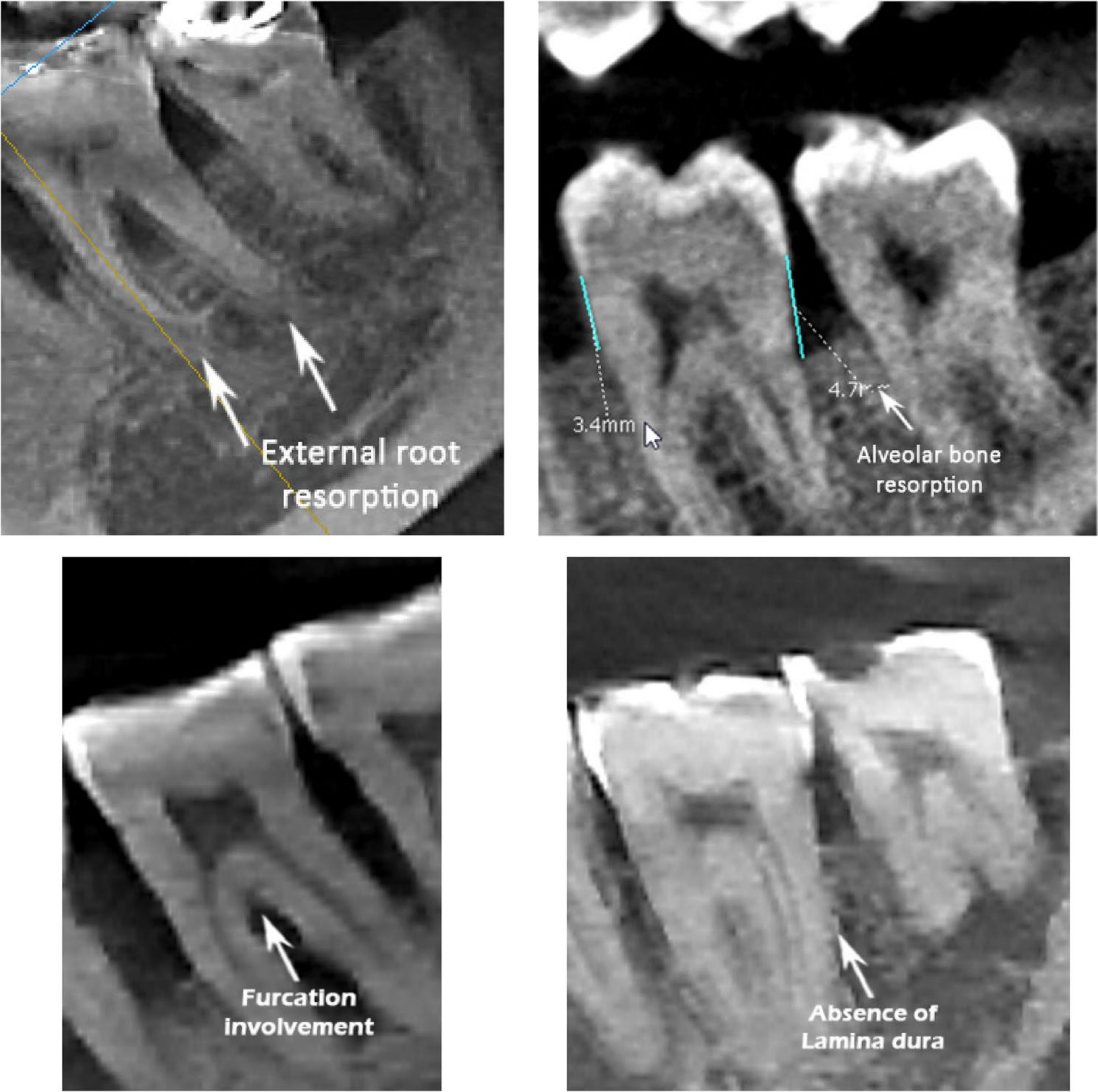
CBCT sagittal sections showing M2’s dental caries, ERR, ABR, FI and the absence of LDs

The study variables include M2’s dental caries, RCT, ERR, FI, ABR, and LD. The presence of dental caries in the molar region was evaluated in axial, sagittal, and coronal views (Fig. 1) by detecting radiolucencies consistent with carious lesions [21, 22]. The presence of RCT indicates that there is radiopaque material present, which can be seen on X-rays, occupying the pulp chamber and/or root canals [23]. The ERR on the distal surface of M2 was evaluated, with a clear loss of substance from the root surface deemed present based on the criteria of established by Al-Khateeb and Bataineh [24]. FI refers to “the pathologic resorption of bone within a furcation” [25] and was assessed through both axial and sagittal observations of defects [26]. Lamina dura (LD) was evaluated in the sagittal view based on the presence or absence of a continuous white line surrounding the M2 root [27]. Representative CBCT images illustrating these pathologies (ERR, FI, ABR, LD absence) are presented in Fig. 2.

### Assessment and measurement

The assessment of variables in this study focused on the presence or absence of dental caries, ERR, FI, and LD in M2s. Additionally, the ABR was evaluated by precisely measuring the alveolar crest height at six anatomical sites around each second molar outlined by Guo et al. [28]. These anatomical sites included:

- Coronally viewed sites: Mid-buccal (B) and mid-lingual (L).
- Sagittally viewed sites: Distobuccal (DB), distolingual/palatal (DL), mesiobuccal (MB), and mesiolingual/palatal (ML).

The alveolar crest height refers to “the distance between the cementoenamel junction and the apical base of the periodontal bone defect” [28].

### Reliability

Measurement reliability was ensured through a structured calibration protocol involving two qualified examiners. The primary investigator (AMA), a periodontist with five years of CBCT interpretation experience, evaluated all 400 cases comprising the final study dataset. Prior to formal assessment, an independent set of 20 calibration cases (distinct from the primary sample) was randomly selected from the same radiology archives. This calibration subset underwent rigorous validation: AMA performed repeat evaluations at one-week intervals for intraobserver agreement, followed by independent assessment by a second examiner (WMS), a board-certified oral radiologist with over ten years of CBCT diagnostic experience. All diagnostic discrepancies were resolved through consensus discussions until unanimous agreement was achieved for all parameters (caries, ERR, FI, ABR, LD). Quantitative analysis using Cohen’s kappa [29] demonstrated excellent agreement (intraobserver: κ = 0.91, 95% CI: 0.85–0.97; interobserver: κ = 0.87, 95% CI: 0.79–0.94). Following calibration, the primary investigator evaluated the 400 study cases without further involvement of the second examiner.

### Statistical analysis

Data were analyzed using SPSS v24 (IBM, Armonk, NY). Frequencies, percentages and means ± SD were calculated. Associations between group and categorical outcomes were tested with the chi-square test. The Mann–Whitney U test compared mean ABR values. A multivariable logistic regression model produced adjusted ORs with 95% CIs for each pathology. Statistical significance was defined as *p* < 0.05.

## Results

Analysis of 400 CBCT scans (mean patient age: 40.56 ± 13.35 years) revealed significant associations between fully erupted M3s and adjacent M2 pathologies. While demographic variations (Table 1) existed—notably higher female proportion in Group II (72.0% vs. 51.0% in Group I) and younger age distribution (73.0% vs. 54.5% aged 18–35 years)—these potential confounders were rigorously addressed through adjustment in regression models. All reported odds ratios (Tables 4 and 5) represent adjusted estimates controlling for sex, age group, side, and arch location.

**Table 1:** Distribution of the sample within the study groups according to sex, side, and age

Variable		Group I		Group II		Total	
		*n*	%	*n*	%	*n*	%
Gender	Male	98	49.0	56	28.0	154	38.5
	Female	102	51.0	144	72.0	246	61.5
Side	Right	99	49.5	91	45.5	190	47.50
	Left	101	50.5	109	54.5	210	52.50
Age	18 to 35 years	109	54.5	146	73.0	255	63.75
	36 to 50 years	78	39.0	37	18.5	115	28.75
	> 50 years	13	6.5	17	8.5	30	7.50
Total		200	100.0	200	100.0	400	100.0

Findings of the chi-square test in Table 2 show that Group I demonstrated substantially higher pathology prevalence than Group II (all *p* < 0.001). Caries affected 71.0% of Group I M2s versus 7.0% in Group II. ERR occurred in 18.5% of Group I cases compared to 3.5%, while FI and RCTs affected 48.5% and 19.0% of Group I M2s respectively (vs. 4.5% and 2.5% in Group II). LD absence was significantly more frequent in Group I (28.0% vs. 3.5%).

**Table 2:** Comparison of dental and periodontal findings in M2s

Variable		Group I		Group II		*P* value
		*n*	%	*n*	%	
Caries	Present	142	71.0	14	7.0	0.000*
	Absent	58	29.0	186	93.0	
ERR	Present	37	18.5	7	3.5	0.000*
	Absent	163	81.5	193	96.5	
FI	Present	97	48.5	9	4.5	0.000*
	Absent	103	51.5	191	95.5	
RCT	Present	38	19.0	5	2.5	0.000*
	Absent	162	81.0	195	97.5	
LD	Present	144	72.0	193	96.5	0.000*
	Absent	56	28.0	7	3.5	

*Abbreviations*: *ERR* External root resorption, *FI* Furcation involvement, *RCT* Root canal treatment, *LD* Lamina dura

*Statistically significant at *p* < 0.05, according to Chi-Square test

Mann-Whitney U tests confirmed significantly elevated ABR at all six sites in Group I (Table3). Mean ABR values in Group I consistently exceeding Group II measurements (all*p*<0.001).

**Table 3:** Comparison of ABR in M2 molars between the study groups

Measurement site	Group I	Group II	*P*-value
	Mean ± SD	Mean ± SD	
MB	3.08 ± 1.82	2.17 ± 0.52	0.000*
ML	3.05 ± 1.71	2.14 ± 0.47	0.000*
B	3.37 ± 1.75	2.25 ± 0.59	0.000*
L	3.57 ± 1.81	2.16 ± 0.47	0.000*
DB	3.55 ± 1.82	2.24 ± 0.52	0.000*
DL	3.29 ± 1.78	2.07 ± 0.51	0.000*

*Abbreviations*:* MB* Mesiobuccal, *ML* Mesiolingual, *B* Mid-buccal, *L* Mid-lingual, *DB* Distobuccal, *DL* Distolingual, *SD* Standard deviation

*Statistically significant at *p* < 0.05, according to Mann-Whitney U test

Two-level multivariable logistic regression models (adjusting for sex, side, arch, and age group) revealed significant demographic associations within Group I (Table 4). Females exhibited 2.89-fold higher caries adjusted odds than males (*p* = 0.001). Left-sided M2s showed increased caries (*p* = 0.005) and RCT risk (*p* = 0.047). Mandibular M2s demonstrated higher caries (*p* = 0.030) and ERR susceptibility (*p* = 0.020). Patients aged 36–50 years had elevated caries (*p* = 0.031), ERR (*p* = 0.045), and FI risks (*p* = 0.020).

**Table 4:** Association of M2 pathologies in group I with other factors

Variable	Caries		ERR		FI		RCT		LD	
	AOR (95% CI)	*p* value	AOR (95% CI)	*p* value	AOR (95% CI)	*p* value	AOR (95% CI)	*p* value	AOR (95% CI)	*p* value
Gender										
Male	1		1		1		1		1	
Female	2.89 (1.52, 5.49)	0.001*	1.122 (0.55, 2.92)	0.751	0.591 (0.34, 1.03)	0.065	1.85 (0.89, 3.82)	0.099	0.78 (0.42, 1.44)	0.420
Side										
Right	1		1		1		1		1	
Left	2.50 (1.32, 4.75)	0.005*	1.49 (0.72, 3.06)	0.285	1.17 (0.67, 2.038)	0.580	2.09 (1.01, 4.33)	0.047*	1.12 (0.61, 2.08)	0.715
Arch										
Maxilla	1		1		1		1		1	
Mandible	1.99 (1.07, 3.73)	0.030*	2.44 (1.15, 5.20)	0.020*	0.96 (0.55, 1.67)	0.887	1.69 (0.82, 3.48)	0.152	1.65 (0.88, 3.08)	0.117
Age										
18–35 years	1		1		1		1		1	
36–50 years	2.08 (1.07, 4.04)	0.031*	2.19 (1.02, 4.69)	0.045*	2.01 (1.12, 3.63)	0.020*	0.76 (0.37, 1.57)	0.456	2.10 (1.07, 4.09)	0.030*
> 50 years	6.96 (0.87, 55.51)	0.067	3.02 (0.82, 11.12)	0.097	2.36 (0.73, 7.70)	0.153	1.16 (0.24, 5.67)	0.854	9.43 (2.65, 33.59)	0.001*

*Abbreviations*: *AOR* Adjusted odds ratio, *95% CI* Confidence interval, *ERR* External root resorption, *FI* Furcation involvement, *RCT* Root canal treatment, *LD* Lamina dura

*Statistically significant at *p* < 0.05, according to multivariable logistic regression controlling for sex, side, arch, and age group

The presence of erupted M3s (Group I) significantly increased the likelihood of adjacent M2 pathologies compared to Group II (Table 5). Adjusted ORs demonstrated substantially higher risks for caries, ERR, FI, RCT, and LD absence (all *p* < 0.001).

**Table 5:** Effect of erupted M3s on adjacent M2 pathologies

Group	Caries		ERR		FI		RCT		LD	
	AOR (95% CI)	*p* value	AOR (95% CI)	*p* value	AOR (95% CI)	*p* value	AOR (95% CI)	*p* value	AOR (95% CI)	*p* value
Group II	1		1		1		1		1	
Group I	32.53 (14.44, 60.66)	0.000*	6.26 (2.72, 14.42)	0.000*	19.99 (9.69, 41.22)	0.000*	9.15 (3.52, 23.78)	0.000*	10.72 (4.78, 24.22)	0.000*

*Abbreviations*: *AOR* Adjusted odds ratio, *95% CI* Confidence interval, *ERR* External root resorption, *FI* Furcation involvement, *RCT* Root canal treatment, *LD* Lamina dura

*Statistically significant at *p* < 0.05, according to multivariable logistic regression controlling for sex, side, arch, and age group

## Discussion

The presence of fully erupted third molars (M3s) has been widely hypothesized to influence periodontal and cariogenic risks in adjacent second molars (M2s) [6, 30, 31]. This study corroborates these associations within the Yemeni population, showing significantly elevated odds of M2 pathologies, including dental caries, ERR, FI, RCT, and LD absence, in cases with erupted M3s (Group I) compared to those without (Group II) (all *p* < 0.001). These findings underscore the necessity for implementing structured radiographic surveillance protocols in clinical practice.

Our findings are consistent with prior research on caries in M2s associated with retained M3s [15, 16]. It was implied that plaque may be undisturbed in the approximal area, which is often inaccessible to cleaning devices [16]. Subsequently, the presence of M3s may lead to the appearance of caries on the distal surface of M2s [32]. A large longitudinal study with a 25-year follow-up reported a relative risk ratio of 2.53, which meant that patients with erupted M3s were at 153% greater risk of caries on the distal surface of M2s in comparison with subjects missing M3s [30].

Importantly, the caries prevalence in Group I (71.0%) greatly exceeded rates reported in prior studies (3.2–52%) [16, 33]. This discrepancy likely reflects both methodological and population-specific factors. While Al-Khateeb and Bataineh [24] reported 21.5% caries prevalence using panoramic radiographs, our CBCT methodology provides superior detection of interproximal and subgingival lesions through three-dimensional reconstruction [34]. Additionally, Yemen’s sociocultural context—characterized by limited preventive care access, high smoking/khat chewing prevalence [17, 18], and dietary patterns—may amplify caries progression. The stark contrast with Group II (7.0% caries prevalence) underscores McArdle and Renton’s [35] assertion that distal M2 caries rarely develop without adjacent M3s. AlHobail et al. [31] further contextualized these findings, suggesting that caries adjacent to erupted M3s often represent late-stage complications linked to long-term oral hygiene challenges.

The significant association between erupted M3s and RCTs (OR = 9.15) aligns with Uysal et al. [36], who identified M3s as endodontic risk factors. This relationship likely stems from caries progression into the pulp complex. Regarding periodontal sequelae, FI—defined as pathological bone resorption into multirooted tooth furcations [37, 38]—exhibited the strongest association with M3 presence (OR = 19.99). This profound bone loss naturally transitions to our ABR findings, where Group I demonstrated significantly greater resorption at all anatomical sites (3.05–3.57 mm vs. 2.07–2.25 mm). These observations corroborate Nunn et al. [30] and extend Li et al.‘s [15] reported ABR risk (OR = 1.77 in their cohort) by quantifying site-specific bone loss magnitudes.

ERR and LD absence represent distinct but interrelated manifestations of periodontal compromise. While ERR (18.5% prevalence in Group I) indicates direct root surface destruction potentially linked to M3-induced biomechanical stresses [39], LD absence (28.0%) reflects disruption of the periodontal ligament-bone interface. Our findings contrast with Li et al. [15], who reported non-significant ERR differences, possibly due to their use of less sensitive 2D radiography versus CBCT [34]. The co-occurrence of these conditions in Group I suggests synergistic pathological processes.

Maxillary M2s exhibited greater ABR than mandibular counterparts across both groups (*p* < 0.001). This disparity likely stems from fundamental anatomical differences in bone density and architecture. The mandible’s characteristically thicker cortical bone structure [40, 41] appears to confer greater resistance to resorptive processes, whereas the maxilla’s predominantly trabecular composition may heighten vulnerability to pathological remodeling under biomechanical stresses from adjacent third molars.

Subgroup analyses further revealed critical demographic influences on pathological manifestations. Regarding age effects, patients over 50 years exhibited a striking 9.43-fold increased risk of LD absence (*p* = 0.001), while those aged 36–50 years showed significantly elevated risks for caries, ERR, and FI compared to younger adults (18–35 years). This pattern likely reflects the cumulative impact of prolonged pathogenic exposure, extended challenges in maintaining posterior segment hygiene, and age-related shifts in bone metabolism that collectively exacerbate periodontal compromise over time.

Concerning sex and side differences, female participants demonstrated a 2.89-fold higher caries risk than males (*p* = 0.001). This complies with Poszytek and Górski [16], where the risk of caries was greater in female patients (*p* < 0.00001). This may be attributed to gender-specific variations in dietary habits, oral hygiene practices, or socioeconomic influences on dental care access. Moreover, sex disparities in caries prevalence (adjusted OR = 2.89 for females) contrast with Kang et al. [32] and Falci et al. [42], possibly due to population-specific variations in oral hygiene awareness and socioeconomic factors. Simultaneously, the observed left-sided predominance in caries and RCTs may reflect Yemen’s culturally established pattern of unilateral khat chewing [43], wherein prolonged substance retention on one side of the dentition creates localized cariogenic environments and complicates hygiene efforts in posterior sextants.

Variations with studies reporting weaker associations [15, 33] may stem from diagnostic methodology. Górski et al.‘s [33] panoramic radiography underestimates ERR by 4.3-fold versus CBCT [34]. While CBCT enabled precise structural assessment, our retrospective design lacks clinical parameters (e.g., probing depths) and direct behavioral data. The young, female-skewed sample (63.75% aged 18–35; 61.5% female) and focus on Yemenis limit generalizability. Future prospective studies should integrate clinical examinations with behavioral assessments to validate these findings across diverse populations.

## Conclusions

This study identifies a significant association between fully erupted M3s and periodontal pathologies in adjacent M2s within the Yemeni population. While radiographic findings suggest elevated risks for caries, ERR, FI, and ABR, the retrospective design precludes causal conclusions. Clinicians should consider vigilant monitoring of M2s adjacent to erupted M3s, particularly in populations with limited access to preventive care. Future prospective studies incorporating clinical examinations and behavioral data (e.g., oral hygiene, khat use) are warranted to validate these associations and inform region-specific guidelines.

## Data Availability

The datasets used and/or analyzed during the current study are available from the corresponding author upon reasonable request.

## Abbreviations

ABR: Alveolar bone resorption
B: Mid-buccal
CBCT: Cone-beam computed tomography
CI: Confidence interval
DB: Distobuccal
DL: Distolingual
ERR: External root resorption
FI: Furcation involvement
L: Mid-lingual
LD: Lamina dura
M2s: Second molars
M3s: Third molars
MB: Mesiobuccal
ML: Mesiolingual
AOR: Adjusted odds ratio
RCT: Root canal treatment
SD: Standard deviation
SPSS: Statistical Package for Social Sciences
